# One-Dimensional Perovskite Manganite Oxide Nanostructures: Recent Developments in Synthesis, Characterization, Transport Properties, and Applications

**DOI:** 10.1186/s11671-016-1320-1

**Published:** 2016-03-01

**Authors:** Lei Li, Lizhi Liang, Heng Wu, Xinhua Zhu

**Affiliations:** National Laboratory of Solid State Microstructures, School of Physics, Nanjing University, Nanjing, 210093 China; State Key Laboratory of Materials-Oriented Chemical Engineering (MCE), Nanjing University of Technology, Nanjing, 210009 China

**Keywords:** Manganites, One-dimensional nanostructures, Synthesis, Characterization, Applications

## Abstract

One-dimensional nanostructures, including nanowires, nanorods, nanotubes, nanofibers, and nanobelts, have promising applications in mesoscopic physics and nanoscale devices. In contrast to other nanostructures, one-dimensional nanostructures can provide unique advantages in investigating the size and dimensionality dependence of the materials’ physical properties, such as electrical, thermal, and mechanical performances, and in constructing nanoscale electronic and optoelectronic devices. Among the one-dimensional nanostructures, one-dimensional perovskite manganite nanostructures have been received much attention due to their unusual electron transport and magnetic properties, which are indispensable for the applications in microelectronic, magnetic, and spintronic devices. In the past two decades, much effort has been made to synthesize and characterize one-dimensional perovskite manganite nanostructures in the forms of nanorods, nanowires, nanotubes, and nanobelts. Various physical and chemical deposition techniques and growth mechanisms are explored and developed to control the morphology, identical shape, uniform size, crystalline structure, defects, and homogenous stoichiometry of the one-dimensional perovskite manganite nanostructures. This article provides a comprehensive review of the state-of-the-art research activities that focus on the rational synthesis, structural characterization, fundamental properties, and unique applications of one-dimensional perovskite manganite nanostructures in nanotechnology. It begins with the rational synthesis of one-dimensional perovskite manganite nanostructures and then summarizes their structural characterizations. Fundamental physical properties of one-dimensional perovskite manganite nanostructures are also highlighted, and a range of unique applications in information storages, field-effect transistors, and spintronic devices are discussed. Finally, we conclude this review with some perspectives/outlook and future researches in these fields.

## Review

One-dimensional perovskite oxide nanostructures, including nanowires, nanorods, nanotubes, nanofibers, and nanobelts, have attracted much attention from scientists for their unique physical properties dependent on the size and dimensionality [1–5]. They are also expected to play important roles as both interconnects and key units in nanoscale electronic, optoelectronic, electrochemical, and electromechanical devices [6–8]. As compared to the zero-dimensional perovskite oxide nanostructures (or quantum dots) and two-dimensional perovskite nanostructures (or quantum wells), the research progress of one-dimensional perovskite oxide nanostructures has been slow until very recently, as hindered from the problems in fabrication and synthesis of these nanostructures with well-controlled dimensions, uniform sizes, phase purity, and homogenous chemical compositions. In recent years, many physical techniques such as advanced nanolithographic techniques (e.g., electron beam or focused ion beam (FIB) writing, proximal probe patterning, AFM lithography, X-ray, or extreme UV lithography) have been developed to fabricate one-dimensional perovskite oxide nanostructures with controllable sizes and morphology [9–14]. In the meantime, chemical synthesis approaches are also developed to synthesize one-dimensional perovskite oxide nanostructures with controllable sizes and morphology, crystalline structures, and chemical compositions, which provide an alternative and intriguing strategy for producing one-dimensional perovskite oxide nanostructures in terms of material diversity, cost, throughput, and the potential advantage of high-volume production [15–18]. Up to date, several reviews on one-dimensional perovskite oxide nanostructures have been published, which provide an overview of research directions in synthesis and applications of one-dimensional perovskite oxide nanostructures [19–22]. For example, the review from Rørvik et al. presents an excellent summary of the current status of one-dimensional perovskite ferroelectric nanostructures [19]. The reviews contributed by Zhu et al. are focused on the synthesis, structural characterization, fundamental physical properties, and applications of perovskite oxide nanotubes and nanowires, respectively [20, 21]. A review on the behavior of one-dimensional perovskite oxide nanostructures, their properties, and the different fabrication approaches to achieve such structures is also available [22]. Among the one-dimensional perovskite oxide nanostructures, one-dimensional perovskite manganite oxide nanostructures (in the forms of nanorods, nanowires, nanotubes, and nanobelts) play important roles in scientific researches and applications in microelectronic, magnetic, and spintronic devices due to their unusual electron transport and magnetic properties. Recently, the research progress on the electronic phase separation in low-dimensional perovskite manganite nanostructures (e.g., nanoparticles, nanowires/nanotubes, and nanostructured films and/or patterns) is reported [23]. In this work, we focus on the recent research activities in the one-dimensional perovskite manganite nanostructures, which provide a comprehensive review of the state-of-the-art of the one-dimensional perovskite manganite nanostructures that covers their synthesis, characterization, transport properties, and applications. Finally, we conclude this article with some perspectives and outlook.

## Synthesis Techniques for One-Dimensional Perovskite Manganite Nanostructures

Up to date, a variety of techniques are developed to fabricate one-dimensional perovskite manganite nanostructures. Basically, these approaches can be classified into two strategies: (i) physical approach and (ii) chemical approach [5–7]. In the physical approach, one-dimensional perovskite manganite nanostructures are patterned from bulk or film counterpart materials by a combination of lithography and etching. The chemical approach, in which one-dimensional perovskite manganite nanostructures are assembled from basic building blocks such as atoms or molecules, much like the way nature uses proteins and other macromolecules to construct complex biological systems, represents a powerful alternative approach to conventional physical methods. In this section, we briefly describe various techniques used for fabricating one-dimensional perovskite manganite nanostructures.

### Physical Approaches

#### Photolithography

Photolithography is an advanced technique to fabricate nanopatterns on substrates coated with polymer materials by mechanical force or sputtering technique. As it can manufacture extensive patterning tautologically, photolithography techniques have the advantages of being practicable, efficient, and economic [24–26]. For example, to understand the role of electronic phase separation (EPS) in the emergent transport behaviors of one-dimensional manganite structures, La_0.33_Pr_0.34_Ca_0.33_MnO_3_ (LPCMO) nanowires were fabricated from a single-crystalline LPCMO thin film via optical lithography, where the width of the manganite wires is reduced to a scale on the order of the inherent phase separation [24]. It is found that by reducing a single-crystal LPCMO thin film to a wire with a width comparable to a scale on the order of the inherent EPS, the system exhibits ultra-sharp jumps in resistivity, and such resistivity jumps are attributed to a reduction of the transport lanes to a single channel. As the insulating barriers of the charge-ordered state are broken by the reduction of temperature or an increase in magnetic field, the resistance in the wire shows sharp jumps around the metal–insulator (M–I) transition, which reflects the nature of the first-order phase transition between ferromagnetic metal and charge-ordered insulator domains [24]. Liu et al. [25] also prepared quasi-one-dimensional oxide nanoconstriction arrays via nanosphere lithography. They dropped a drop of aqueous suspension of SiO_2_ microspheres, with a diameter of 1.5 μm, onto a SrTO_3_ (100) substrate. These microspheres could self-assemble during the drying process and finally turned into a hexagon-like ordered monolayer. Then, a reactive ion etching process was proceeded to reduce the sizes of the microspheres. Subsequently, the substrate was put into a pulsed laser deposition (PLD) chamber for the deposition of La_0.67_Sr_0.33_MnO_3_; after that, the sample was transferred into a furnace and annealed at 750 °C. After removing the microspheres, a La_0.67_Sr_0.33_MnO_3_ nanoconstriction array was obtained. Under the low oxygen pressure, the La_0.67_Sr_0.33_MnO_3_ film was deposited with the oxygen deficiency in La_0.67_Sr_0.33_MnO_3_ nanoconstriction, the sample had to be further annealed at 900 °C for 8 h in air. Finally, the La_0.67_Sr_0.33_MnO_3_ size of nanoconstriction obtained was around 100 nm. Peña et al. [26] fabricated La_2/3_Sr_1/3_MnO_3_ microbridges by another method. They first deposited La_2/3_Sr_1/3_MnO_3_ films with a thickness range from 15 to 50 nm onto SrTiO_3_ substrates by RF sputtering. Then, the films were patterned into microbridges with different sizes by using standard photolithographic techniques.

#### Focused Ion Beam Milling

Recently, large aspect-ratio (length-to-width >300) single-crystal nanowires of La_2/3_Ca_1/3_MnO_3_ were also fabricated by combined optical and FIB lithographies, which preserved their functional properties [27]. Remarkably, an enhanced magnetoresistance value of 34 % in an applied magnetic field of 0.1 T in the narrowest 150-nm nanowire was obtained. Such behavior is ascribed to the strain release at the edges together with a destabilization of the insulating regions. This opens new strategies to implement these structures in functional spintronic devices. FIB is also used to fabricate manganite oxide nanobridges [28–31]. For example, Singh-Bhalla et al. [28, 29] fabricated (La_0.5_Pr_0.5_)_0.67_Ca_0.33_MnO_3_ nanobridges and microbridges with a width ranging from 100 nm to 1 μm. They first deposited single-crystalline, epitaxial 30-nm-thick (La_0.5_Pr_0.5_)_0.67_Ca_0.33_MnO_3_ films on the NdGaO_3_ (110) substrates at 820 °C by PLD. Then, a combination of photolithography and a FIB was employed to fabricate the (La_0.5_Pr_0.5_)_0.67_Ca_0.33_MnO_3_ nanobridges and microbridges. Pallecchi et al. [30] deposited La_0.7_Sr_0.3_MnO_3_ films on SrTiO_3_ (001) substrates by pulsed laser ablation and obtained La_0.7_Sr_0.3_MnO_3_ narrow channels with widths of 0.2–1.0 μm by a Ga^+^ FIB. Céspedes et al. [31] also patterned a manganite nanobridge by FIB. They grew La_0.7_Sr_0.3_MnO_3_ films by PLD. Then, a focused Ga^+^ beam was used to fabricate a La_0.7_Sr_0.3_MnO_3_ nanobridge with dimensions of less than 20 nm.

#### Electron Beam Lithography

Electron beam lithography (EBL) is a nanofabrication technique in rapid development [32]. Guo et al. [32] grew La_0.67_Ca_0.33_MnO_3_ films with thickness of ~100 nm on SrTiO_3_ (100) substrates by a PLD technique and fabricated La_0.67_Ca_0.33_MnO_3_ microbridges with different widths (e.g., 1.5 μm, 1 μm, and 500 nm) by EBL technology. Beekman et al. [33] also grew thinner La_0.7_Ca_0.3_MnO_3_ films (with a thickness range of 20–70 nm) on SrTiO_3_ (001) substrates by DC sputtering. And then, they fabricated a La_0.7_Ca_0.3_MnO_3_ microbridge with a width of 5 μm by using EBL technology and Ar etching.

### Chemical Approaches

#### One-Dimensional Perovskite Manganite Oxide Nanostructures Synthesized by Hydrothermal Process

A hydrothermal method is a common method to fabricate manganite nanowires, which usually involves heating an aqueous suspension of precursor in a Teflon vessel at befitting temperatures and pressures. A mineralizer which is conducive to the crystallization is generally injected to control the morphology of products [34–37]. For example, Zhu et al. [34] used KMnO_4_, MnCl_2_·4H_2_O, La(NO_3_)_3_·6H_2_O, Ba(OH)_2_·8H_2_O, and Sr(NO_3_)_2_ as raw materials and KOH as a mineralizer to synthesize La_0.5_Ba_0.5_MnO_3_ nanowires. The reaction reagents were dissolved into deionized water to form a solution, to which KOH was added with stirring to adjust the alkalinity of the solution. The aqueous solution was reacted at 270 °C for 25 h to get La_0.5_Ba_0.5_MnO_3_; furthermore, another crystallization reaction occurred at 280 °C for 50 h to get La_0.5_Sr_0.5_MnO_3_ nanowires. The nanowire diameters were in the range of 30–150 nm for La_0.5_Ba_0.5_MnO_3_ and 50–400 nm for La_0.5_Sr_0.5_MnO_3_. By the same method, Datta et al. [35] also synthesized the single-crystalline La_0.5_Sr_0.5_MnO_3_ nanowires with a diameter of ~50 nm and a length up to 10.0 μm. It was found that these La_0.5_Sr_0.5_MnO_3_ nanowires had a ferromagnetic–paramagnetic transition temperature (Curie temperature, *T*_C_) at around 325 K, which was very close to the bulk value (~330 K) of the single crystal with the same composition. It was also found that the functional behavior was likely to be retained even after the diameter size of the nanowires was reduced to 45 nm. The electrical transport measurements on a single nanowire demonstrated that the nanowire exhibited an insulating behavior within the measured temperature range, which was similar to the bulk system. Single-crystalline La_0.5_Ca_0.5_MnO_3_ nanowires with lengths ranging from several to several tens of micrometers and a uniform diameter of ~80 nm were also grown by a hydrothermal method [36]. The La_0.5_Ca_0.5_MnO_3_ nanowires had an orthorhombic perovskite structure with very clean surfaces and grew along the (100) direction. An enhanced *T*_C_ was observed in these nanowires, which was ascribed to the unit cell contraction and anisotropy. Rao et al. [37] also reported the hydrothermal synthesis of the charge-ordering Pr_0.5_Ca_0.5_MnO_3_ (PCMO) single-crystalline nanowires with a diameter of ~50 nm and a length of a few microns. They found that in these PCMO nanowires, the charge-ordered phase was weakened and the antiferromagnetic phase disappeared, whereas a ferromagnetic phase was observed in this one-dimensional manganite oxide nanowire.

#### One-Dimensional Perovskite Manganite Nanostructures Synthesized by Template Assistance

Template synthesis of manganite nanotubes and nanowires is a versatile and inexpensive technique, which combines a sol–gel process and the use of porous sacrificial substrates of either silicon or alumina as templates [38–45]. The size, shape, and structural properties of the assembly are simply controlled by the templates used, which form ordered arrayed nanowires and nanotubes. As a result, the diameter and length of the nanowires/nanotubes are corresponded closely to the pore. By this method, Li et al. [38] synthesized the LPCMO/MgO core–shell nanowires with diameters about tens of nanometers in two steps. First, chemical vapor deposition was used to grow MgO nanowires on the MgO (100) substrates coated with Au nanoparticles. Then, the LPCMO shell layers were deposited on the MgO nanowires by a PLD process. They finally obtained the LPCMO/MgO core–shell nanowires with diameters of 30 nm and lengths in a range of several micrometers to tens of micrometers. Similarly, Beltran-Huarac et al. [39] grew bamboo-like carbon nanotubes (BCNTs) and then deposited the La_0.67_Sr_0.33_MnO_3_ films onto the bamboo-like carbon nanotubes. Finally, they obtained one-dimensional La_0.67_Sr_0.33_MnO_3_/BCNTs with diameters ranging from 100 to 160 nm and lengths over 10 μm. Atalay et al. [40] used Ca(NO_3_)_2_·*X*H_2_O, La(NO_3_)_3_·6H_2_O, and Mn(NO_3_)_2_·*X*H_2_O as raw materials and ethylene glycol as a solvent. The solution with a pH value of 2–6 was stirred in high temperature until a gel was formed. Then, the gel was filled into a porous anodized aluminum oxide (AAO) template. Finally, the template was annealed at 400 °C for 2 h and at 700 °C for 2 h with a rate of 2 °C/min. After dissolution in 1 M NaOH, the nanowires with a diameter of 185–195 nm were obtained. Similarly, Ma et al. [41] also used an AAO template and a sol–gel process to fabricate La_0.8_Ca_0.2_MnO_3_ nanowires with a diameter of 30 nm. Carretero-Genevrier et al. [42] produced single-crystalline La_0.7_Sr_0.3_MnO_3_ nanowires by polymer template-directed chemical solution synthesis. In addition, perovskite rare-earth manganese tubes such as La_0.67_Sr_0.33_MnO_3_, La_0.67_Ca_0.33_MnO_3_, and La_0.325_Pr_0.300_Ca_0.375_MnO_3_ are also fabricated using a sol–gel template synthesis process [43, 44]. Their typical length was about 6 to 8 μm, and the average wall thickness is 45, 60, and 150 nm for La_0.67_Sr_0.33_MnO_3_, La_0.67_Ca_0.33_MnO_3_, and La_0.325_Pr_0.300_Ca_0.375_MnO_3_, respectively. The walls of the tubes were composed of magnetic nanograins, and their sizes are less than the critical size for multidomain formation in manganites. As a consequence, each particle that constituted the nanotube walls was a single magnetic domain. The La_0.6_Sr_0.4_CoO_3_ nanotubes with a diameter of 100 nm and the nanowires with a diameter of 40–60 nm were also formed by shaping the sol with the cylinder pores in an AAO template [45].

#### One-Dimensional Perovskite Manganite Nanostructures Produced from Electrospinning Process

Electrospinning is a time and cost-effective technique that produces ultra-fine fibers with diameters in the dozens-of-nanometers range through the action of an external electric field imposed on a precursor solution [46–48]. Hayat et al. [46] added barium acetate and manganese acetate tetrahydrate into acetic acid separately followed by stirring for 10–15 min, then poured the barium acetate and manganese acetate solution into the mixture of ethanol and polyvinyl pyrrolidone, and stirred them at room temperature for 16 h. The applied electric voltage between the needle tip and the collector was 10 kV. Finally, the collected nanofibers were heated at 600 °C for 2 h. Nanofibers with an average diameter of less than 100 nm were obtained. Jugdersuren et al. [47] dissolved La(NO_3_)_3_·6H_2_O, Mn(NO_3_)_2_·2H_2_O, and Sr(NO_3_)_2_ into water and added polyvinyl pyrrolidone (PVP) beads to bind the solution. Then, the electrospinning process proceed; after that, nanowires were collected and annealed at 550 °C for 3 h in argon and 3 % hydrogen gas mixture, additionally at 730 °C for 1 h in argon and 10 % oxygen atmosphere. Finally, they got La_0.67_Sr_0.33_MnO_3_ nanowires with diameters in a range of 80–300 nm and length in 200 μm. Zhou et al. [48] obtained thinner LaMnO_3_ nanofibers (50–100 nm) by a semblable way using lanthanum acetate and manganese acetate as raw materials and an applied electric voltage of 15 kV between the collector and the needle tip.

## Structural Characterization of One-Dimensional Perovskite Manganite Nanostructures

Contemporarily, all kinds of techniques have been used to characterize the nanostructures of one-dimensional manganite, like X-ray diffraction (XRD), scanning electron microscope (SEM), transmission electron microscopy (TEM), selected area electron diffraction (SAED), and so on. Moreover, some techniques such as electron energy loss spectroscopy (EELS) and energy-dispersive X-ray spectrometer (EDX) can be utilized to analyze the chemical composition [20, 21].

### Manganite Nanowires/Nanorods

**Fig. 1:**
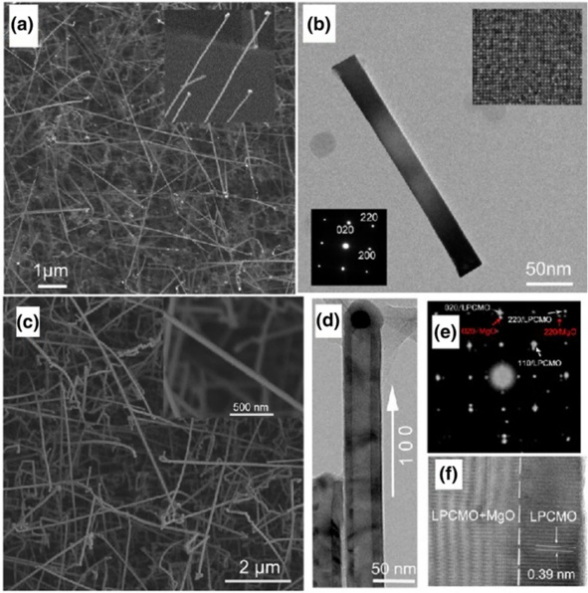
**a** SEM image of the MgO nanowires on the MgO substrate. The *inset* is the enlarged view. The *light spots* are Au nanoparticles. **b** TEM image of an individual MgO nanowire. The *upper right and bottom left insets* show the corresponding HRTEM image and SAED pattern, respectively. **c** SEM image of the LPCMO/MgO nanowires on the MgO substrate. The *inset* is the enlarged view. **d** TEM image of the LPCMO/MgO nanowires. **e** SAED pattern and **f** HRTEM image of an individual LPCMO/MgO nanowire (reproduced with permission of [38])

**Fig. 2:**
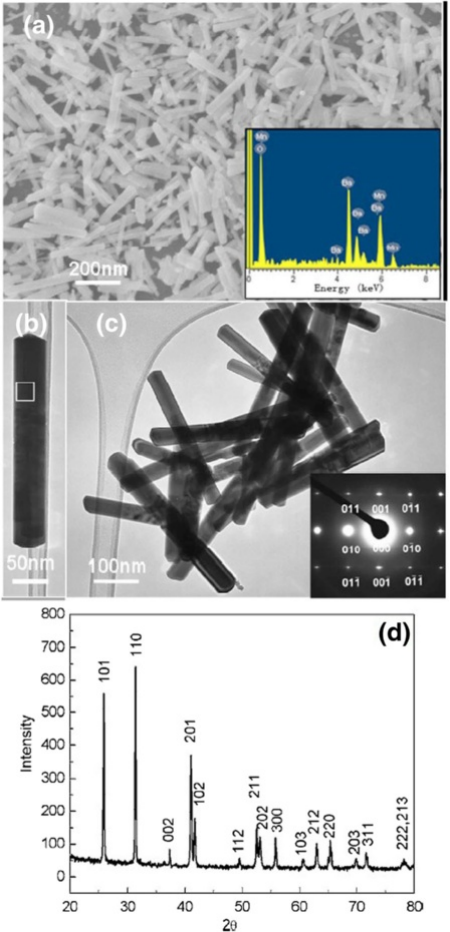
**a** SEM, **b** and **c** TEM images, and **d** XRD pattern of BaMnO_3_ nanorods. The *insets* in **a** and **c** are EDX and SAED patterns, respectively (reproduced with permission of [49])

**Fig. 3:**
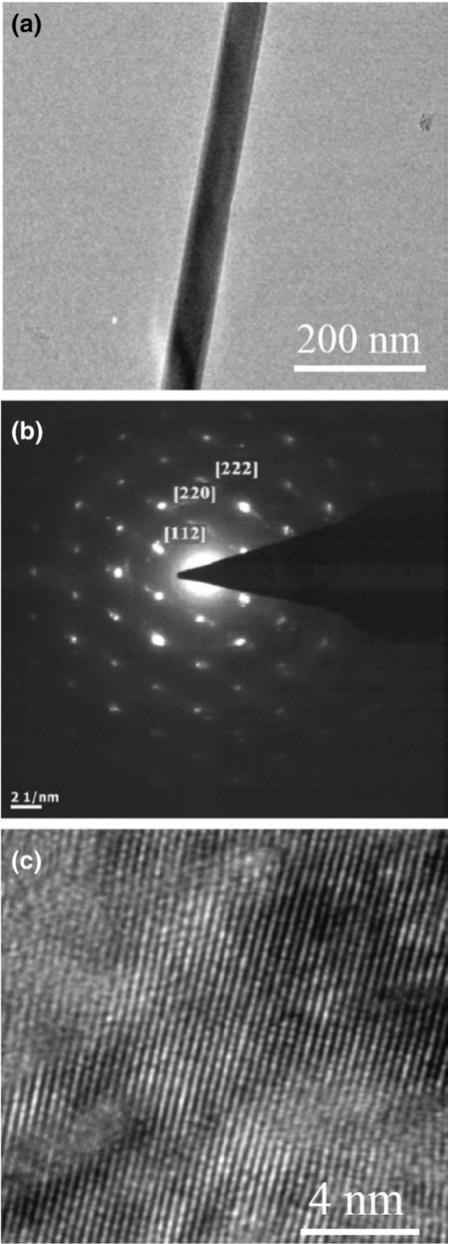
**a** TEM image of a single nanowire, **b** selected area diffraction pattern of the nanowire, and **c** HRTEM image of the nanowire (reproduced with permission of [35])

**Fig. 4:**
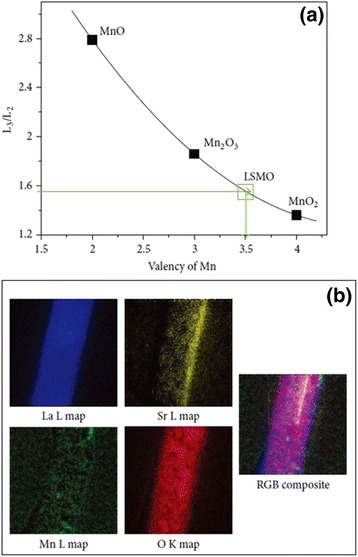
**a** Intensity ratios of L_3_ and L_2_ lines of different Mn oxide compounds as a function of their known valency. **b** EFTEM image of a La_0.5_Sr_0.5_MnO_3_ nanowire (reproduced with permission of [35])

Figure 1a shows the microstructural characterization of MgO nanowires and La_0.33_Pr_0.34_Ca_0.33_MnO_3_/MgO core–shell nanowires synthesized by a confinement growth method [38]. Figure 1a, c shows the SEM images of the MgO nanowires and the La_0.33_Pr_0.34_Ca_0.33_MnO_3_/MgO core–shell nanowires, respectively. From Fig. 1a, it can be seen that the top of the nanowires is capped by the Au nanoparticles. Figure 1b, d shows the TEM images of an individual MgO nanowire and the LPCMO/MgO nanowires, respectively. It can be inferred that the diameter of a single MgO nanowire is about 30 nm, and the thickness of LPCMO shell is about 10 nm. The SAED pattern and high-resolution TEM (HRTEM) image, as seen in Fig. 1e, f, reveal good epitaxial growth of the LPCMO shell on the MgO core. Wang et al. [49] synthesized BaMnO_3_ nanorods by the composite-hydroxide-mediated method, as shown in Fig. 2. Figure 2a–c demonstrates the BaMnO_3_ nanorods with a diameters of 20–50 nm and lengths of 150–250 nm, which belong to a hexagonal structure with lattice parameters of *a* = 5.699 Å and *c* = 4.817 Å, as proven by the XRD pattern (Fig. 2d). The structural and elemental characterizations were also performed on the single-crystalline La_0.5_Sr_0.5_MnO_3_ nanowires synthesized by a hydrothermal method [35]. The TEM image of a single nanowire is shown in Fig. 3a. The single-crystalline nature of the nanowires was confirmed from the diffraction pattern and HRTEM images, as shown in Fig. 3b, c, respectively. The lattice spacing was around 0.311 nm, and the (*hkl*) values were estimated from diffraction pattern data. The TEM-EDAX data also revealed that the atomic percentage ratio of (La+Sr):Mn:O was approximately 1:1:3, which was the desired composition. Therefore, the nanowires fabricated are pure phase and single crystalline in nature. The elemental analysis of the La_0.5_Sr_0.5_MnO_3_ nanowires was done by EELS on different single nanowires repeatedly, and the valency of Mn was estimated from the calibration curve shown in Fig. 4a. From the calibration curve, the valency of Mn in the La_0.5_Sr_0.5_MnO_3_ nanowires was estimated to be ~3.5, which was very close to its bulk value. Energy-filtered TEM (EFTEM) image was used to check the homogeneity of the elemental distribution within each nanowire. Figure 4b shows the EFTEM image of a La_0.5_Sr_0.5_MnO_3_ nanowire, where red, green, blue, and yellow colors are used for elements O, Mn, La, and Sr, respectively. The EFTEM analysis shows that all the constituent elements La, Sr, Mn, and O are homogeneously distributed within the La_0.5_Sr_0.5_MnO_3_ nanowire.

**Fig. 5:**
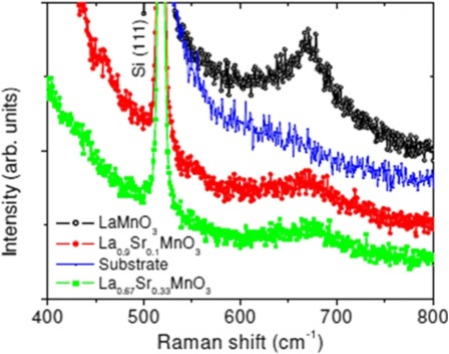
Raman spectra of the La_1 − *x*_Sr_*x*_MnO_3_ (*x* = 0, 0.10, and 0.33) nanowires (reproduced with permission of [47])

To obtain the structural information of the manganite nanowires from vibrational spectra, Raman scattering investigations were also performed. For example, Jugdersuren et al. [47] reported the Raman spectra of the La_1 − *x*_Sr_*x*_MnO_3_ (*x* = 0, 0.10, and 0.33) nanowires, as shown in Fig. 5. A broad peak appeared at 660 cm^−1^ in the Raman spectra of LaMnO_3_, which was identified as the B_2g_ peak corresponding to the stretching and bending of the MnO_6_ octahedra. For the La_0.9_Sr_0.1_MnO_3_ and La_0.67_Sr_0.33_MnO_3_, the B_2g_ peak shifted to around 670 cm^−1^ with decreasing intensity.

### Manganite Nanotubes

**Fig. 6:**
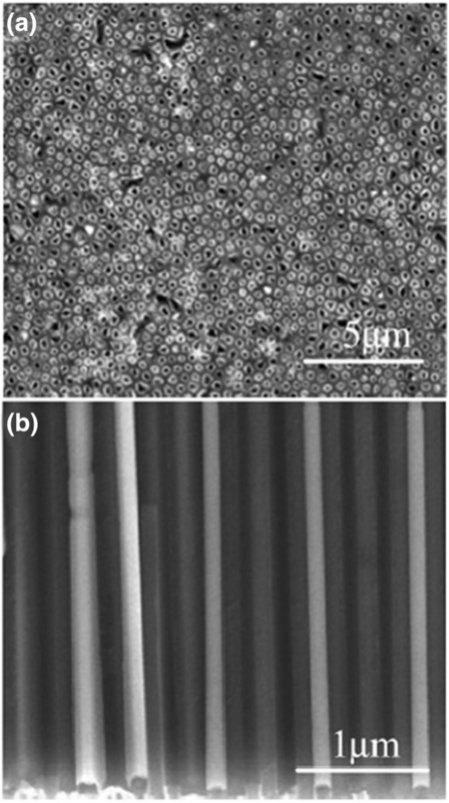
**a** Top view and **b** cross-sectional SEM micrographs of La_0.7_Ca_0.3_MnO_3_ nanotube arrays (reproduced with permission of [50])

**Fig. 7:**
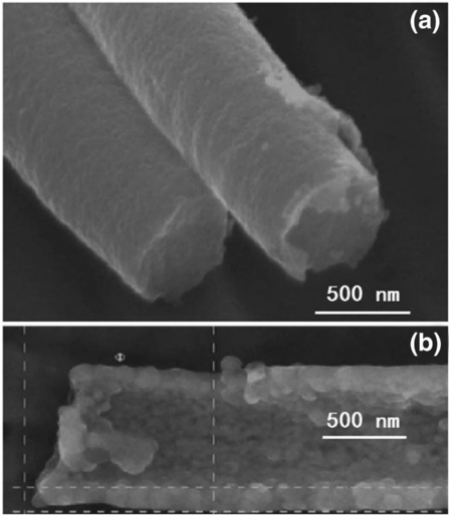
SEM micrograph of **a** La_0.325_Pr_0.300_Ca_0.375_MnO_3_ nanotubes and **b** a broken tube of La_0.325_Pr_0.300_Ca_0.375_MnO_3_ (reproduced with permission of [51])

Kumaresavanji et al. [50] synthesized highly ordered La_0.7_Ca_0.3_MnO_3_ nanotube arrays by a template-assisted sol–gel method. Figure 6 shows the top view and cross-sectional SEM micrographs of La_0.7_Ca_0.3_MnO_3_ nanotube arrays. The diameters of the nanotubes were around 190 ± 10 nm, and the average length of the nanotubes was about to 60 μm, corresponding to the pore diameters and thickness of the template. Manganite nanotubes can be prepared not only by AAO template assistance; plastic templates such as porous polycarbonate films are also widely used. Figure 7a, b shows the SEM micrographs of La_0.325_Pr_0.300_Ca_0.375_MnO_3_ nanotubes grown in the polycarbonate template with a pore size of 1 μm [51]. The nanotubes had an average diameter of 800 nm and a length of about 4 μm, about half of the template thickness. Figure 7b shows a cross-section view of a broken tube with 150-nm-thick walls. It can be distinguished that the walls are formed by nanoparticles with a size of about 50 nm. Similarly, Wang et al. [45] also reported the La_0.6_Sr_0.4_CoO_3_ nanotubes with a diameter of 100 nm and the nanowires with a diameter of 40–60 nm formed by shaping the sol into the cylinder pores in an AAO template. The difference between the diameters of the nanowires and the nanotubes may be due to their different shrinkage directions. For the nanowire, the shrinkage was towards the center of the cross section of the nanowires, whereas the shrinkage was towards the walls of the membrane pores in the case of nanotubes. The nanowires were tens of microns long, which were longer than the nanotubes.

### Manganite Nanofibers/Nanobelts

**Fig. 8:**
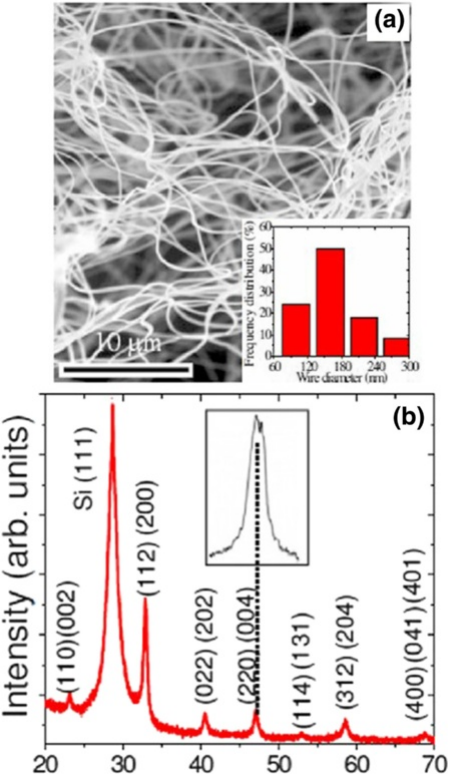
**a** SEM images of La_0.67_Sr_0.33_MnO_3_ wires. The *inset* shows the frequency distribution of the wire diameter. **b** XRD pattern of as-grown La_0.67_Sr_0.33_MnO_3_ wires (reproduced with permission of [47])

**Fig. 9:**
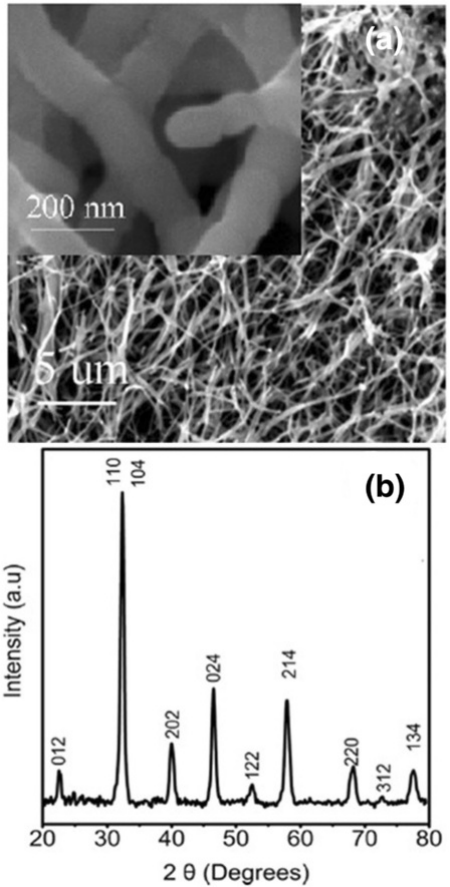
**a** SEM images and **b** XRD pattern of La_0.8_Sr_0.2_MnO_3_ nanofibers. The *inset* in **a** is the enlarged view (reproduced with permission of [52])

The morphology of the as-grown La_0.67_Sr_0.33_MnO_3_ wires by the electrospinning process was examined by SEM, and the crystal structure was determined by XRD [47]. A typical SEM image of the as-grown La_0.67_Sr_0.33_MnO_3_ wires grown on a silicon substrate is shown in Fig. 8a. The inset of Fig. 8a shows the frequency distribution of the wire diameter. The diameter of the La_0.67_Sr_0.33_MnO_3_ wires was in a range of 80–300 nm and lengths up to 200 μm. Figure 8b demonstrates the XRD pattern of the La_0.67_Sr_0.33_MnO_3_ wires, indicating that all the XRD peaks can be indexed to an orthorhombic (Pnma) perovskite structure without any other secondary phases. The inset of Fig. 8b displays the peak splitting corresponding to the orthorhombic phase. Zhi et al. [52] synthesized the La_0.8_Sr_0.2_MnO_3_ nanofibers by an electrospinning process. As seen in Fig. 9, the lengths of nanofibers are up to tens of micrometers and 150–225 nm in diameter. Their XRD peaks are indexed with the La_0.8_Sr_0.2_MnO_3_ rhombohedral structure.

### Manganite Nanobridges and Microbridges

**Fig. 10:**
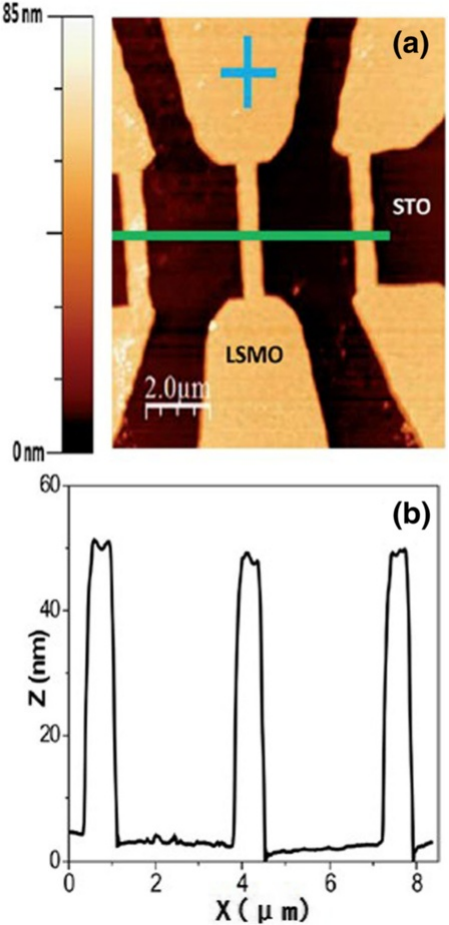
**a** Topographic image of a lithographed La_2/3_Sr_1/3_MnO_3_ thin film and its **b** relief profile (reproduced with permission of [26])

**Fig. 11:**
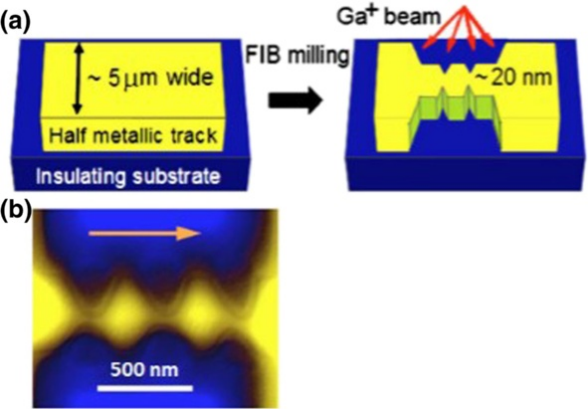
**a** Schematic of the fabrication process. **b** SEM of an La_0.7_Sr_0.3_MnO_3_ triple nanoconstriction (reproduced with permission of [31])

**Fig. 12:**
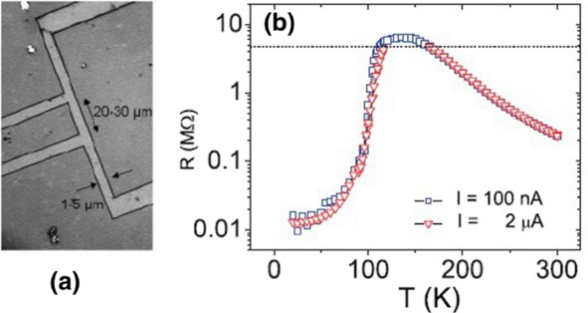
**a** An electron microscopy picture of a typical microbridge. **b** *R*(*T*) at two different current densities (reproduced with permission of [33])

Nanobridge structures have attracted much interest due to their special structures and physical properties. Figure 10a shows the topographic image of La_2/3_Sr_1/3_MnO_3_ microbridges (with a height of ~45 nm) synthesized by photolithographic techniques [26], and Fig. 10b shows the relief profile of the microbridges measured along the green line shown in Fig. 10a. The image indicates that the engraving process employed in the experiment reaches the STO substrate surface; in other words, the bridges are electrically and positionally isolated from each other. Figure 11 shows a unique La_0.7_Sr_0.3_MnO_3_ nanobridge with single and multiple nanoconstrictions synthesized by FIB [31]. Figure 11a shows the schematic of the FIB milling of a double nanoconstriction departing from a 5.0-μm track on 50–150-nm films. Figure 11b shows the SEM image of a triple nanoconstriction with a width of 20–50 nm. A domain wall is supposed to form at the nanoconstriction. Recently, La_0.67_Ca_0.33_MnO_3_ microbridges with different widths are also fabricated from the well epitaxial La_0.67_Ca_0.33_MnO_3_ films by EBL technology [32]. An anomalous positive magnetoresistance effect was observed in these La_0.67_Ca_0.33_MnO_3_ microbridges. The underlying mechanism for this phenomenon is the confined geometry, which is dominated by the filamentary conduction mechanism. The magnetoresistance of the microbridges also shows an interesting behavior for the enhanced *e*–*e* interactions in the presence of spin disorder. It can be decreased and can even change its sign in the bridges with widths of 1.5 and 1.0 μm under magnetic field of 1 T. Figure 12a shows an electron microscopy picture of a typical La_0.7_Ca_0.3_MnO_3_ microbridge patterned by EBL [33]. The bridge has a four-point configuration with a width of 5 μm and 20–30 μm between the voltage contacts. The temperature dependence of resistance of a bridge measured on a flat STO at two different current densities is shown in Fig. 12b (squares for *J* = 2 × 10^6^ A/m^2^, triangles for *J* = 4 × 10^7^ A/m^2^), and both curves show a clear M–I transition temperature at *T*_MI_ = 130 K.

## Fundamental Transport Properties of One-Dimensional Perovskite Manganite Oxide Nanostructures

One-dimensional perovskite manganite nanostructures are attractive for their size and dimensionality dependence of physical properties such as electrical and magnetic performances. These properties make it widely used for applications in microelectronic, magnetic, and spintronic devices. In this section, we introduce the electrical and magnetic transport properties of one-dimensional perovskite manganite nanostructures [20, 21].

### Electrical Transport Properties

**Fig. 13:**
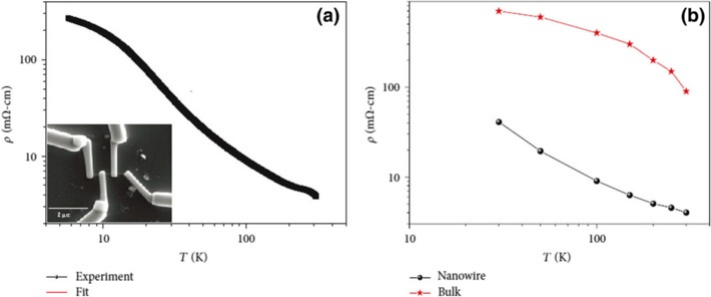
**a** Temperature-dependent resistivity of one single La_0.5_Sr_0.5_MnO_3_ nanowire, measured in the temperature range from 5 to 310 K. The *inset* is the SEM image of the four-wire electrical contact made of Pt patterned on a single 45-nm La_0.5_Sr_0.5_MnO_3_ nanowire. **b** Quantitative comparison of resistivity of La_0.5_Sr_0.5_MnO_3_ nanowires and bulk (reproduced with permission of [35])

**Fig. 14:**
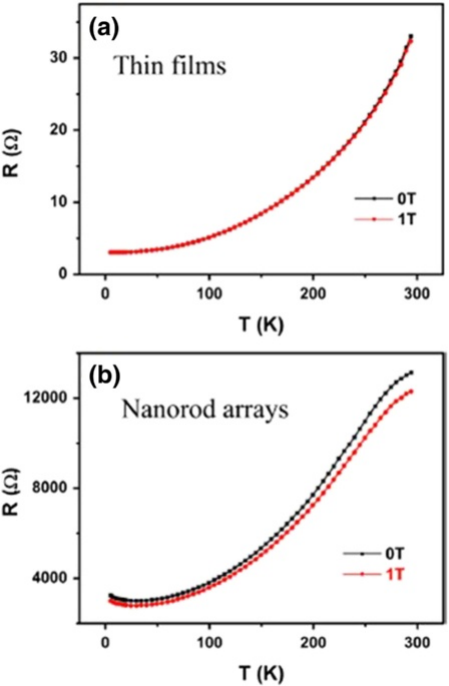
Temperature-dependent resistance for the **a** La_0.67_Sr_0.33_MnO_3_ thin films and the **b** La_0.67_Sr_0.33_MnO_3_/MgO nanorod arrays, respectively, at various magnetic fields (reproduced with permission of [53])

**Fig. 15:**
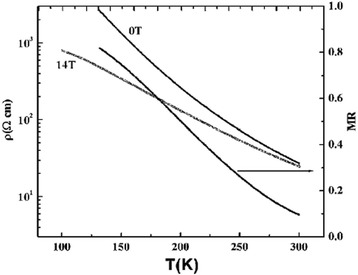
The temperature dependence of resistivity measured under magnetic fields of 0 and 14 T, respectively, and the MR under a field of 14 T (reproduced with permission of [36])

**Fig. 16:**
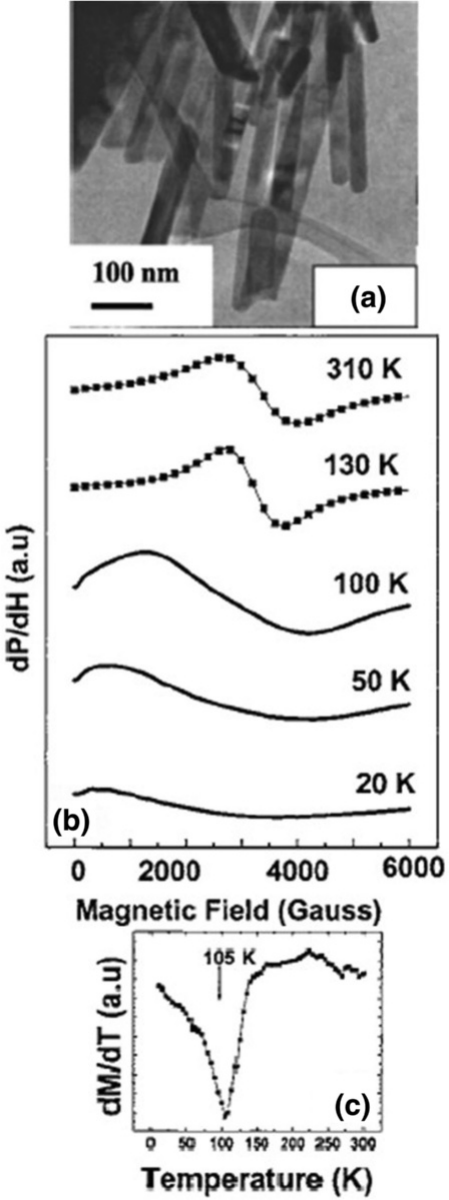
**a** TEM images, **b** EMR signals, and **c** *dM*/*dT* versus *T* of Pr_0.5_Ca_0.5_MnO_3_ nanowires (reproduced with permission of [37])

The temperature variation of resistivity is an important measurement item for the study of electrical transport properties of one-dimensional perovskite manganite nanostructures [35–37]. Figure 13 [35] shows (a) the temperature dependence of resistance for one La_0.5_Sr_0.5_MnO_3_ single nanowire and (b) the quantitative comparison of resistivity of La_0.5_Sr_0.5_MnO_3_ nanowire and bulk prepared by a ceramic method. The inset in the bottom left corner of Fig. 13a shows the SEM image of the four-wire electrical contact made of Pt patterned on a single 45-nm La_0.5_Sr_0.5_MnO_3_ nanowire. From Fig. 13, we can see the insulating behavior of La_0.5_Sr_0.5_MnO_3_ nanowire. The resistivity of La_0.5_Sr_0.5_MnO_3_ bulk is quite higher than that of La_0.5_Sr_0.5_MnO_3_ nanowire. Li et al. [53] compared electrical properties of the simultaneously grown La_0.67_Sr_0.3_MnO_3_/MgO core–shell nanorod arrays and La_0.67_Sr_0.3_MnO_3_ thin films. Figure 14 shows the temperature dependence of resistance for the La_0.67_Sr_0.3_MnO_3_ thin films and La_0.67_Sr_0.3_MnO_3_/MgO nanorod arrays. The resistance of nanorod arrays is about 3 orders higher than that of thin films, due to the enhanced scattering at the grain boundaries. Moreover, a valley is appeared in the curve of nanorod arrays, which is ascribed to a Kondo-type scattering from blocked Mn spins at the grain boundaries. Zhang et al. [36] synthesized La_0.5_Ca_0.5_MnO_3_ (~80 nm) nanowires by a hydrothermal method and studied the transport properties of the nanowires. Figure 15 shows the temperature dependence of resistivity measured under magnetic fields of 0 and 14 T, respectively, and the magnetoresistance (MR) under a magnetic field of 14 T. The MR ratio is defined as MR = [*R*(0) − *R*(*H*)] / *R*(0), where *R*(0) and *R*(*H*) are the resistance in the absence and presence of an external field, respectively. There is no M–I transition observed under two magnetic fields; also, the MR ratio increases with the decreasing temperature. These results are ascribed to the grain boundaries, near which strong spin-dependent scattering of carriers exists, leading to a manganite state, and the line shapes of electron magnetic resonance (EMR) signals change. That is ascribed to the appearance of exchange and anisotropy fields in the ferromagnetic state [54]. Figure 16 shows the TEM images, EMR signals, and *dM*/*dT* versus *T* of Pr_0.5_Ca_0.5_MnO_3_ nanowires with diameters of ~50 nm synthesized by a hydrothermal method [37]. It can be seen from Fig. 16b that symmetric Lorentzian signals become broad and asymmetric below about 130 K, which coheres with the *T*_C_ ~105 K obtained from Fig. 16c.

**Fig. 17:**
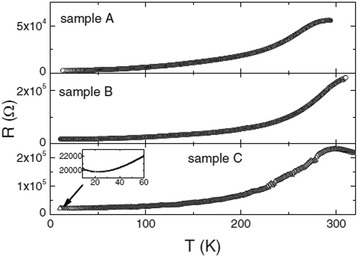
The temperature dependence of resistance of samples A, B, and C (reproduced with permission of [30])

Pallecchi et al. [30] fabricated three kinds of La_0.7_Sr_0.3_MnO_3_ micro/nanobridges (with different widths) by different pattern technologies. Sample A is patterned by optical lithography and wet etching in HCl with a width of ~15 μm, sample B is patterned by AFM local anodization with a width of ~5 μm, and sample C is patterned by FIB milling with a width of ~0.5 μm. Their temperature dependences of resistance of three samples mentioned above are shown in Fig. 17. Sample B has the largest M–I transition temperature (*T*_MI_, above 310 K), which indicates that sample B has better structural quality and optimal oxygen stoichiometry [30]. In the La_0.67_Ca_0.33_MnO_3_ microbridges with different widths fabricated by EBL technology, their magnetoresistance shows an interesting behavior due to the enhanced *e*–*e* interactions in the presence of spin disorder. It can be decreased and even changed its sign in the widths of 1.5- and 1.0-μm bridges under the magnetic field of 1 T, whereas under high magnetic fields of 4 and 7 T, the *e*–*e* interactions are weakened by other interactions, and the positive magnetoresistance is masked by the negative magnetoresistance [32].

### Magnetic Transport Properties

**Fig. 18:**
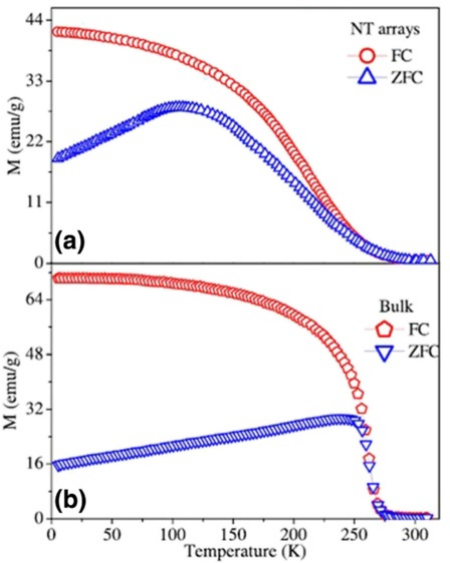
Temperature-dependent magnetization at 100 Oe for **a** La_0.7_Ca_0.3_MnO_3_ nanotube arrays and **b** its bulk counterpart (reproduced with permission of [50])

**Fig. 19:**
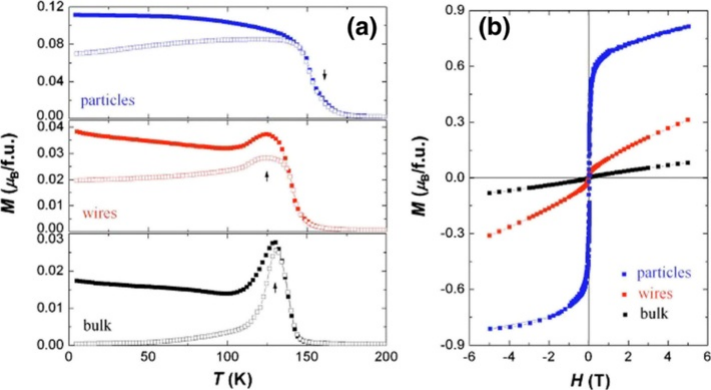
**a** FC (*closed symbols*) and ZFC (*open symbols*) magnetizations for the samples under a field of 1000 Oe. The *arrows* indicate *T*_CO_ (for the bulk and nanowires) or *T*_C_ (for the nanoparticles). **b** *M*–*H* curves for the samples at 5 K (reproduced with permission of [55])

**Fig. 20:**
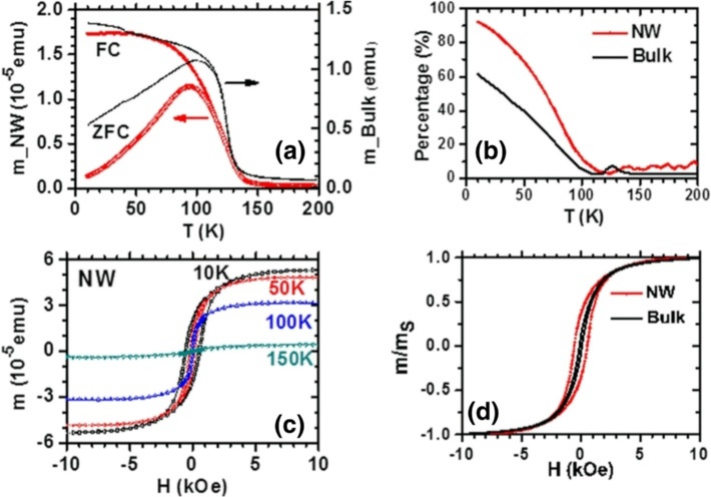
**a** Magnetic moment versus temperature of the LPCMO/MgO nanowires (*NW*) and the LPCMO bulk polycrystalline sample after ZFC and FC. The cooling field and the measuring field are both 200 Oe. **b** The percentage of the frozen phase defined as [*m*(FC) − *m*(ZFC)] / *m*(FC), **c** the field-dependent magnetic moment of the LPCMO/MgO nanowires at different temperatures, and **d** the hysteresis loops of the nanowires and the bulk sample measured at *T* = 10 K (reproduced with permission of [38])

Kumaresavanji et al. [50] reported the magnetic properties of La_0.7_Ca_0.3_MnO_3_ nanotube arrays and its counterpart. Figure 18 shows temperature-dependent magnetization of La_0.7_Ca_0.3_MnO_3_ nanotube arrays and its bulk counterpart. The Curie temperature (*T*_C_) is over a broad temperature at 236 K for nanotube arrays and sharp at 258 K for a bulk sample. A furcation between field-cooled (FC) and zero-field-cooled (ZFC) curves is observed below the *T*_C_. Wang and Fan [55] measured the different magnetic properties of the Ca_0.82_La_0.18_MnO_3_ nanowires, nanoparticles, and bulk, as shown in Fig. 19. Figure 19a shows the FC and ZFC temperature-dependent magnetization for the samples. The charge-ordered (CO) temperature (*T*_CO_) is 132 K for bulk and ~124 K for nanowires. But the nanoparticles exhibit a ferromagnetism (*T*_C_ ~165 K), in which the CO disappears. Figure 19b shows the *M*–*H* curves measured at 5 K, indicating an FM behavior in the nanoparticles and an AFM behavior in the bulk. The nanowires exhibit an AFM state with slight ferromagnetism. Li et al. [38] also reported the magnetic transport properties of the single-crystalline LPCMO nanowires with diameters about tens of nanometers, as shown in Fig. 20. In Fig. 20a, the ZFC curve and the FC curve of the LPCMO nanowires are split at a blocking temperature of *T*_b_ = 93 K when the temperature is decreased. Such a ZFC/FC deviation is very similar to that of the bulk polycrystalline LPCMO sample also shown in Fig. 20a and is due to the frozen of the magnetic moment. The differences between the ZFC and FC magnetic moments in the nanowire, defined as the frozen phase magnetic moment, is significantly larger than that in the bulk counterpart below the blocking temperature sample, as shown in Fig. 20b. In bulk or thin-film LPCMO, the frozen phase is generally regarded to be related to the phase competition between the FM metallic phase and the AFM–CO phase [56]. So, in the nanowires, the increased amount of frozen phase concentration implies the stronger phase competition in the low-dimensional system. Figure 20c, d displays the magnetic field dependence of the magnetic moments of the LPCMO nanowires and the bulk counterpart. As observed in Fig. 20c, both the saturation magnetic moment (*m*_s_) and the coercivity (*H*_c_) in the LPCMO nanowires were increased as the temperature was decreased, which was similar to that in bulk or thin-film manganites. However, the differences between the nanowire and the bulk sample were also observed. The *H*_c_ value of the LPCMO nanowires was much larger than that of the LPCMO bulk sample. For example, at *T* = 10 K, *H*_c_ is about 550 Oe in the nanowire but only about 100 Oe in the bulk sample as shown in Fig. 20d. The larger *H*_c_ in the nanowires could be attributed to their stronger domain wall pinning at the boundaries of the separated AFM and FM phases caused by the EPS in the nanowires [57]. The above observations suggest that the EPS with a stronger phase competition exists in the one-dimensional structures.

## Applications of One-Dimensional Perovskite Manganite Nanostructures

### Information Storages

One-dimensional manganite oxide has an attractive commercial value for the adhibition in information storages. Among sundry manganites, perovskite manganite is of particular importance due to its colossal magnetoresistance (CMR) [26]. Peña et al. [26] investigated the electronic transport properties of La_2/3_Sr_1/3_MnO_3_ microfabricated bridges. The local *I*–*V* curves are nearly symmetric in the ON state (prior to the transition between low resistance and high resistance states) but present a rectifying performance in the OFF state (high resistance state, HRS). A metal–insulator–metal (M–I–M) geometry with an interface-switching mechanism is introduced: the topmost unit cells of the La_2/3_Sr_1/3_MnO_3_ are the I layer and the remaining La_2/3_Sr_1/3_MnO_3_ layer and scanning probe microscope (SPM) conducting tip which act as the M layer. This geometry allows the research of the electronic properties of the HRS and their dependence on temperature and magnetic field for the first time. The results indicate that the electronic effects are the biggest contributor in the charge depletion, even though mobile ions or ionic defects might affect the current transport. These results represent an important step forward to the oxide-based memory devices.

### Field-Effect Transistors

**Fig. 21:**
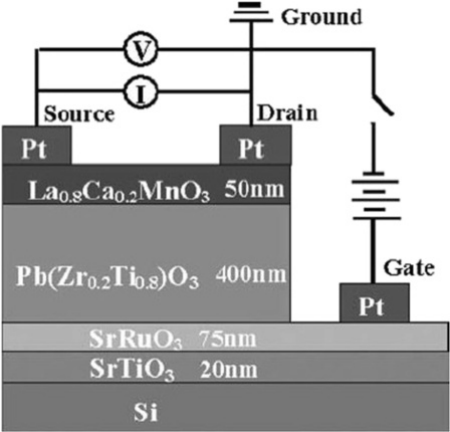
Schematic structure of the FeFET (reproduced with permission of [58])

Field-effect transistor is the simplest form of transistor, which is widely used in large-scale integration. It controls the conductive ability of solid materials by electric field effect. Zhao et al. [58] fabricated an all-perovskite ferroelectric field-effect transistor (FeFET) structure with a Pb(Zr_0.2_Ti_0.8_)O_3_ gate and a CMR La_0.8_Ca_0.2_MnO_3_ channel on Si, as seen in Fig. 21. A maximum modulation of 20 % after an electric field poling of 1.5 × 10^5^ V/cm and 50 % under a magnetic field of 1 T was observed near the M–I transition temperature of the La_0.8_Ca_0.2_MnO_3_ channel. The results demonstrate the feasibility of integrating the CMR-based FeFET on Si.

### Spintronic Devices

In the one-dimensional perovskite manganite oxide nanostructures, their magnetic, electronic, and lattice degrees of freedom interact with each other through double exchange and Jahn–Teller interaction, leading to delicate unbalances between the magnetic, electronic, and lattice degrees of freedom in these materials at the nanoscale, and thus, new outstanding properties can be achieved [59]. Therefore, one-dimensional perovskite manganite nanostructures are viewed as functional building blocks for the transport of charge and spins for the assembly of electronic, magnetic, and sensing devices [60, 61]. By using manganite oxide nanowires as building blocks, one can create manganite oxide nanowire-based lateral spin valves or magnetic tunneling junction (MTJ) devices. A possible scheme starts with a bottom-up synthesized FM manganite oxide nanowire, and a small portion of which is be converted into nonmagnetic (NM) by using selective Ar^+^ ion milling, leading to an FM–NM–FM lateral device. Such a manganite oxide nanowire-based spintronic device is attractive, whereas the centrally modified region must be thin enough to retain the spin coherent transport. The enhanced low-field MR [47, 62] and the Curie temperature [63] and significant magnetic anisotropy [42] have also been observed in the perovskite manganite oxide nanowires. In addition, the morphology of manganite oxide nanowire can be also controlled by annealing or growing on engineered substrates, which could significantly affect their physical properties [60, 64]. Therefore, such large MR and the great tunability of one-dimensional perovskite manganite nanostructures are much attractive for spintronic applications. By using La_0.67_Sr_0.33_MO and SrTiO_3_ as FM and insulating layer, respectively, all-oxide MTJ devices were first fabricated by Lu et al. [65] and Sun et al. [66]. Furthermore, a record tunneling magnetoresistance ratio of 1850 % was also reported by Bowen et al. [67]. Despite these promising results have been made, the working temperature of all the perovskite oxide-based MTJ devices remains lower than the room temperature, which is the major issue to be resolved before commercial applications of all perovskite oxide-based MTJ devices.

## Conclusions

In summary, this article provides a comprehensive review on recent developments in synthesis, characterization, transport properties, and applications of one-dimensional manganite oxide nanostructures (including nanorods, nanowires, nanotubes, and nanofibers). Nowadays, one-dimensional manganite oxide nanostructures are widely used for applications in nanostructure-based devices because of their fascinating electrical and magnetic transport properties. Although many exciting progresses and potential applications of one-dimensional manganite oxide nanostructures have been made, considerable challenges remain to be resolved. In terms of the fabrication techniques, the top-down (physical approach) fabrication technique often requires expensive equipment and complicated processing and also usually faces the challenge of structural defects (such as edge roughness) in the lithographically patterned one-dimensional manganite oxide nanostructures. In addition, most of the perovskite manganite oxide materials need high deposition temperature and the typical electron beam and photo resists are incompatible with this requirement. The bottom-up (chemical approach) synthesis of one-dimensional manganite oxide nanostructures with precise and reproducible controls in composition, morphology, and physical properties is challenging for one-dimensional manganite oxide nanostructures used for spintronic devices. In terms of microelectronic devices, there have been some revolutionary breakthroughs in spintronics, such as spin valves, magnetic tunneling junctions, and spin field-effect transistors. However, several problems for one-dimensional manganite oxide nanostructures used for spintronic devices remain unresolved and some technical challenges lie ahead. For example, the spin polarization of manganite oxides decays rapidly with temperature, and the working temperature of the one-dimensional manganite oxide nanostructure-based spintronic device is often lower than the room temperature. The low-dimensional spin-dependent transport exists in the one-dimensional manganite oxide nanostructures, and the physical properties of the interfaces within one-dimensional manganite oxide nanostructure-based devices remain elusive. Furthermore, the defect chemistries and the stoichiometry-property correlations in the one-dimensional perovskite manganite oxide nanostructures are quite complex. In addition, new device processing techniques are also urgent to be developed. With the researches into one-dimensional manganite oxide nanostructures spreading their wings and becoming more extensive, it is expected that the fascinating achievements towards the practical applications of one-dimensional manganite oxide nanostructures in the fields of microelectronics, magnetics, and spintronics will be made. An exciting new era for the applications of one-dimensional manganite oxide nanostructures in oxide microelectronics is on the horizon!
